# Risk, treatment duration, and recurrence risk of postpartum affective disorder in women with no prior psychiatric history: A population-based cohort study

**DOI:** 10.1371/journal.pmed.1002392

**Published:** 2017-09-26

**Authors:** Marie-Louise H. Rasmussen, Marin Strøm, Jan Wohlfahrt, Poul Videbech, Mads Melbye

**Affiliations:** 1 Department of Epidemiology Research, Statens Serum Institut, Copenhagen, Denmark; 2 Center for Neuropsychiatric Depression Research, Mental Health Center Glostrup, Glostrup, Denmark; 3 Department of Medicine, Stanford University School of Medicine, Stanford, California, United States of America; University of Manchester, UNITED KINGDOM

## Abstract

**Background:**

Some 5%–15% of all women experience postpartum depression (PPD), which for many is their first psychiatric disorder. The purpose of this study was to estimate the incidence of postpartum affective disorder (AD), duration of treatment, and rate of subsequent postpartum AD and other affective episodes in a nationwide cohort of women with no prior psychiatric history.

**Methods and findings:**

Linking information from several Danish national registers, we constructed a cohort of 457,317 primiparous mothers with first birth (and subsequent births) from 1 January 1996 to 31 December 2013 (a total of 789,068 births) and no prior psychiatric hospital contacts and/or use of antidepressants. These women were followed from 1 January 1996 to 31 December 2014. Postpartum AD was defined as use of antidepressants and/or hospital contact for PPD within 6 months after childbirth. The main outcome measures were risk of postpartum AD, duration of treatment, and recurrence risk. We observed 4,550 (0.6%) postpartum episodes of AD. The analyses of treatment duration showed that 1 year after the initiation of treatment for their first episode, 27.9% of women were still in treatment; after 4 years, 5.4%. The recurrence risk of postpartum AD for women with a PPD hospital contact after first birth was 55.4 per 100 person-years; for women with postpartum antidepressant medication after first birth, it was 35.0 per 100 person-years. The rate of postpartum AD after second birth for women with no history of postpartum AD was 1.2 per 100 person-years. After adjusting for year of birth and mother’s age, women with PPD hospital contact after first birth had a 46.4 times higher rate (95% CI 31.5–68.4) and women with postpartum antidepressant medication after their first birth had a 26.9 times higher rate (95% CI 21.9–33.2) of a recurrent postpartum episode after their second birth compared to women with no postpartum AD history. Limitations include the use of registry data to identify cases and limited confounder control.

**Conclusions:**

In this study, an episode of postpartum AD was observed for 0.6% of childbirths among women with no prior psychiatric history. The observed episodes were characterized by a relatively short treatment duration, yet the women had a notably high rate of later AD and recurrent episodes of postpartum AD. The recurrence risk of postpartum AD was markedly higher among women with PPD hospital contact after first birth compared to women with postpartum antidepressant medication after first birth. Our results underline the necessity of measures targeted at specific vulnerable groups, such as women who experience PPD as a first psychiatric episode.

## Abstract 2

In a population-based study, Marie-Louise Rasmussen and colleagues examine risk and recurrence of postpartum affective disorder in women with no prior psychiatric history in Denmark.

## Author summary

**Why was this study done?:**

- Postpartum depression (PPD) is a non-psychotic depressive episode occurring in the period following delivery of a child. Depending on the inclusion criteria, it affects 5%–15% of all women after childbirth, which makes it one of the most common postnatal complications of childbearing. Left untreated, the disorder can have long-term implications for both mother and child, including impairment of the child’s development and increased risk of long-term maternal depression.
- Our aim was to describe the risk of postpartum affective disorder (AD) (a broader category that includes PPD) among women with no prior psychiatric episodes, as well as the recurrence risk and the duration of treatment.

**What did the researchers do and find?:**

- We linked data from the Danish national registries and constructed a cohort of 457,317 primiparous mothers with first birth (and subsequent births) from 1 January 1996 to 31 December 2013 (a total of 789,068 births) and no prior psychiatric hospital contacts and/or use of antidepressants.
- In this study, an episode of postpartum AD was observed for 0.6% of childbirths among women with no prior psychiatric history. The observed episodes were characterized by a relatively short treatment duration, yet the women had a notably high rate of later AD and recurrent episodes of postpartum AD. The recurrence risk of postpartum AD was markedly higher among women with a PPD hospital contact after first birth than among women with postpartum antidepressant medication use after first birth.

**What do these findings mean?:**

- These population-based figures provide valuable guidance to physicians treating women with PPD. The study documents the existence of a group of patients who experience elevated rates of subsequent depression and PPD following an initial PPD episode. It underlines the seriousness of single initial episodes and highlights the necessity of both primary and secondary preventive measures.
- Limitations include the use of registry data to identify cases, as this may result in a group of women likely to be at the more severe end of the PPD spectrum; thus, this study does not necessarily account for the milder cases.

## Introduction

Postpartum depression (PPD) is a nonpsychotic depressive episode occurring in the period following delivery of a child. Depending on, for example, the inclusion criteria and the quality of follow-up of women who have given birth, it is reported to affect 5%–15% of all women after childbirth [1,2], which makes it one of the most common postnatal complications of childbearing. Left untreated, the disorder can have long-term implications for both mother and child, including impairment of the child’s development [3–5] and increased risk of long-term maternal depression [6]. A number of different risk factors for PPD have been identified, of which the majority are antenatal, personal, and psychosocial factors [3,7–9]. However, such factors can account for at most a third of the variance in the diagnosis of PPD [10,11], which could indicate a genetic predisposition, as suggested in some studies [12].

Although evidence exists that there is significant heterogeneity in the timing and persistence of maternal depressive symptomatology [13], very few previous studies have distinguished between women with a prior history of psychiatric disease and women with no such history. This lack of differentiation might partly explain the divergence in findings of etiological studies of PPD and in the observed frequency of the disorder, and adds to the ongoing dispute as to whether PPD is a specific disease entity [14]. Additionally, there is a lack of population-based studies investigating the duration of treatment of PPD [15]. It is our assumption that the PPD phenotype among women with no prior psychiatric history is more homogeneous than the phenotype among women with prior psychiatric history. Thus, the main focus of this study was on women with no prior psychiatric history.

The purpose of this study was, by use of Danish national healthcare and population registers, to describe the risk of postpartum affective disorder (AD) among women with no prior psychiatric disorders, the recurrence risk, as well as the duration of treatment in this group.

## Methods

In accordance with Danish law, the use of the register-based data in the study was approved by the Danish Data Protection Agency (no. 2008-54-0472). The study is reported as per STROBE guidelines (S1 STROBE Checklist). A detailed analysis plan was not written prior to the initiation of the project. However, based on the objectives of this study, we defined all basic analyses to be undertaken in meetings with all involved parties (epidemiologists, clinician, and statisticians) prior to the receipt of the registry data and before the start of the analyses. We did not depart from the analysis plan built during these meetings but added post hoc sensitivity analyses as presented. However, in response to review comments, postpartum antidepressant treatment was divided post hoc into postpartum medication and PPD hospital contact.

### Study cohort

Using the Danish Civil Registration System, we established a cohort comprising all women born in Denmark who delivered their first live-born singleton child between 1 January 1996 and 31 December 2013 (*n =* 457,317 women). The Danish personal identification number permits complete follow-up of all persons living in Denmark and accurate linkage of individual-level information from Denmark’s many mandatory national population-based registers. The registries used for this study are described in detail in S1 Text. Women with antidepressant use (ATC: N06) registered in the Danish National Prescription Registry (DNPR) and women registered in the Psychiatric Central Research Register (PCRR) or the National Patient Registry (NPR) with mental illnesses (ICD-8: 29, 30; ICD-10: F0–F9) any time prior to their first delivery were excluded from the cohort. Complete nationwide data on prescriptions were available in the DNPR starting in 1995, so cohort inclusion began 1 January 1996 in order to have information on antidepressant use in the year before delivery in those delivering in early 1996. Follow-up ended 31 December 2014 to include the postpartum period for women delivering in 2013.

### Antidepressant treatment—In general and postpartum

In this study, we defined AD as use of antidepressant medication and/or hospital contact for depression (in- and outpatient). Episodes of postpartum AD were defined as episodes occurring within 6 months after delivery, and all other episodes were referred to as non-postpartum AD. Women with AD were identified based on information in the NPR, the PCRR, and the DNPR. Episodes in the DNPR were identified as women filling at least 1 prescription for antidepressant medication (ATC: N06). Episodes in the NPR and PCRR were identified as women having an in- or outpatient contact for a depressive episode, using main diagnoses only (ICD-8: 2960, 2962, 2968, 2969, 2980, 3004, 3011; ICD-10: F320–F329). For simplicity, outpatients were also referred to as being admitted and discharged from hospitals, e.g., in the analysis of duration of treatment. In the analysis of the risk of postpartum AD and the duration of treatment, we did not discriminate as to whether the episodes were defined by use of medication or hospital contact. In the analysis of the recurrence of AD, we separately analyzed use of postpartum medication and PPD hospital contact after first birth. We interpreted the 2 measures as treatment for the same overall disorder—although they may represent different levels of severity of the disorder.

Initiation of treatment was defined as the date of first filled prescription for an antidepressant medication or first hospital admission date, whichever came first. Period of usage was estimated based on information in the DNPR on number of defined daily doses in the prescription. For refills of prescriptions, a gap of up to 3 months between the calculated last date of usage of a prescription and the dispensing date of the next prescription was permitted to allow for differences in drug intake and prescriber habits. If the gap between prescriptions exceeded 3 months, treatment was considered discontinued, and a new prescription after that was defined as a new incident episode. Likewise, a 3-month cutoff was applied after the discharge date of hospitalizations for defining a new incident episode.

### Statistical analyses

Relative risks of postpartum AD according to parity, year of birth, and mother’s age were estimated by a log-linear binomial regression model—the estimates are mutually adjusted.

The proportion of primiparous women still in treatment by the number of months since the initiation of treatment was estimated by a Kaplan–Meier analysis. Only women with a postpartum episode after their first birth were included in this analysis. Women were included from the first prescription date or first admission date, whichever came first, and were followed until the first of the following events: discontinuation of treatment, second birth, death, emigration, or end of follow-up. This means that, for example, women giving birth in 2012 were followed until the end of 2014 (end of follow-up) unless any of the other above-mentioned events occurred prior to end of follow-up.

Rates of postpartum AD after second birth and rates of non-postpartum AD (after first or second birth) in women with and without a postpartum AD episode after their first birth were estimated as number of episodes divided by the number of person-years, calculated separately based on the number of years since latest childbirth. We used a log-linear Poisson regression model to estimate rate ratios (RRs) for non-postpartum AD in (1) women with a postpartum AD episode after first birth defined by antidepressant medication and (2) women with a postpartum AD episode after first birth defined by hospital contact compared to (3) women with no postpartum AD after first birth. We compared the rate of postpartum and non-postpartum AD in second-time mothers for these same 3 groups. Women with both postpartum antidepressant medication and PPD hospital contact after first birth were classified as PPD hospital contact cases at the time of the first event, regardless of chronological order of the medication prescription and the hospital contact. The RR analyses were adjusted for year of birth and mother’s age. Primiparous women with postpartum AD were followed for non-postpartum AD after end of treatment, whereas follow-up for primiparous women with no postpartum AD episode began 6 months after the birthdate of their firstborn. If women gave birth a second time, they changed status and contributed with person-years to the second-birth analyses. Follow-up for all women continued until the first of the following events: an AD episode, a psychiatric diagnosis other than depression, death, emigration, or end of follow-up. Thus, at any point in the study, no woman, regardless of parity, had any prior history of psychiatric disorders other than a possible postpartum AD after first birth.

Estimation in the supplementary analyses (S2 Text) was performed by the same means as in the main analyses.

## Results

### Risk of postpartum AD

Between 1 January 1996 and 31 December 2013, 457,317 women had a first live-born child. Of these, 273,195 women delivered a second child, and 78,556 women had 3 or more children during the follow-up period. The proportion of women with a history of postpartum AD and the proportion of women with a postpartum AD episode are given in Table 1. Overall, 0.6% (*n =* 4,550) of childbirths were followed by a postpartum AD episode; 2,389 of these episodes occurred in primiparous women. The proportion of postpartum AD episodes increased markedly over the study period, and the risk of an episode was significantly higher among young mothers: the relative risk for mothers <25 years versus mothers 29–31 years was 1.8 (95% CI 1.6–2.0).

**Table 1:** Risk of postpartum affective disorder (AD)—Distribution of number of births, women with prior history of postpartum AD, and postpartum AD episodes according to parity, year of birth, and age.

Category	Number of births	Percent of women with history of postpartum AD (*n*)	Percent of women with a postpartum AD episode (*n*)	Relative Risk^a^ (95% CI)
**All births**	789,068	0.1 (1,102)	0.6 (4,550)	
**Parity**				
1st birth	457,317	—	0.6 (2,389)	1 (ref.)
2nd birth	273,195	0.3 (743)	0.6 (1,680)	1.2 (1.1–1.3)
3+ birth	78,556	0.5 (359)	0.6 (481)	1.2 (1.1–1.3)
**Year of birth**				
1996–2000	179,849	0.03 (46)	0.3 (609)	0.5 (0.4–0.5)
2001–2004	191,965	0.1 (184)	0.6 (1,074)	0.8 (0.8–0.9)
2005–2008	197,011	0.2 (342)	0.7 (1,374)	1.1 (1.0–1–1)
2009–2013	220,243	0.2 (530)	0.7 (1,493)	1 (ref.)
**Mother’s age (years)**				
<25	129,512	0.1 (99)	0.8 (1,075)	1.8 (1.6–2.0)
25–28	215,954	0.1 (248)	0.6 (1,225)	1.2 (1.1–1.3)
29–31	194,251	0.2 (291)	0.5 (1,019)	1 (ref.)
32–34	140,721	0.2 (268)	0.5 (680)	0.9 (0.8–1.0)
35+	108,630	0.2 (196)	0.5 (551)	0.9 (0.8–1.0)

Singleton births from 1996–2013 in Denmark in women with no prior psychiatric events.

^a^Adjusted for parity, year of birth, and mother’s age.

### Duration of treatment

**Fig 1:**
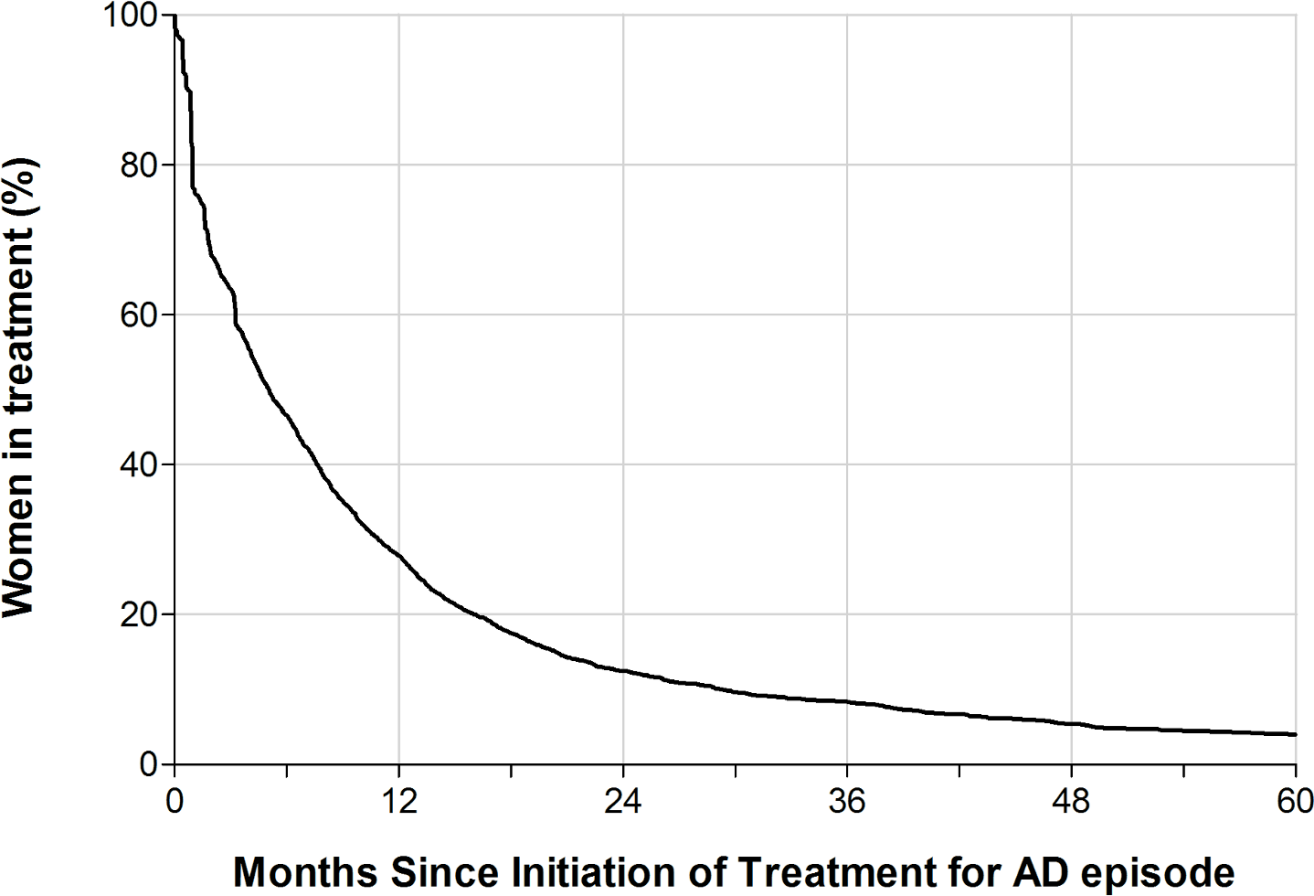
The estimated proportion of women in antidepressant treatment by number of months since the initiation of treatment for a postpartum episode of affective disorder (AD). Primiparous Danish women with a postpartum AD, 1996–2013, with no prior psychiatric disorders.

Fig 1 shows the estimated proportion of women still in treatment for a postpartum episode by the number of months since the initiation of treatment among primiparous women (*n =* 2,389). One year after the first dispensing of antidepressants or hospital contact, 27.9% of the women were still in treatment; after 4 years, 5.4% remained in treatment. A relatively large number of women filled only 1 prescription of antidepressants or were admitted only a short period of time, causing a large decrease in treated women (23%) in the first month. In a sensitivity analysis that included only women filling at least 2 prescriptions or being hospitalized, this decrease in treated women following the first treatment period was 19%.

### Recurrence risk and risk of non-postpartum AD

**Fig 2:**
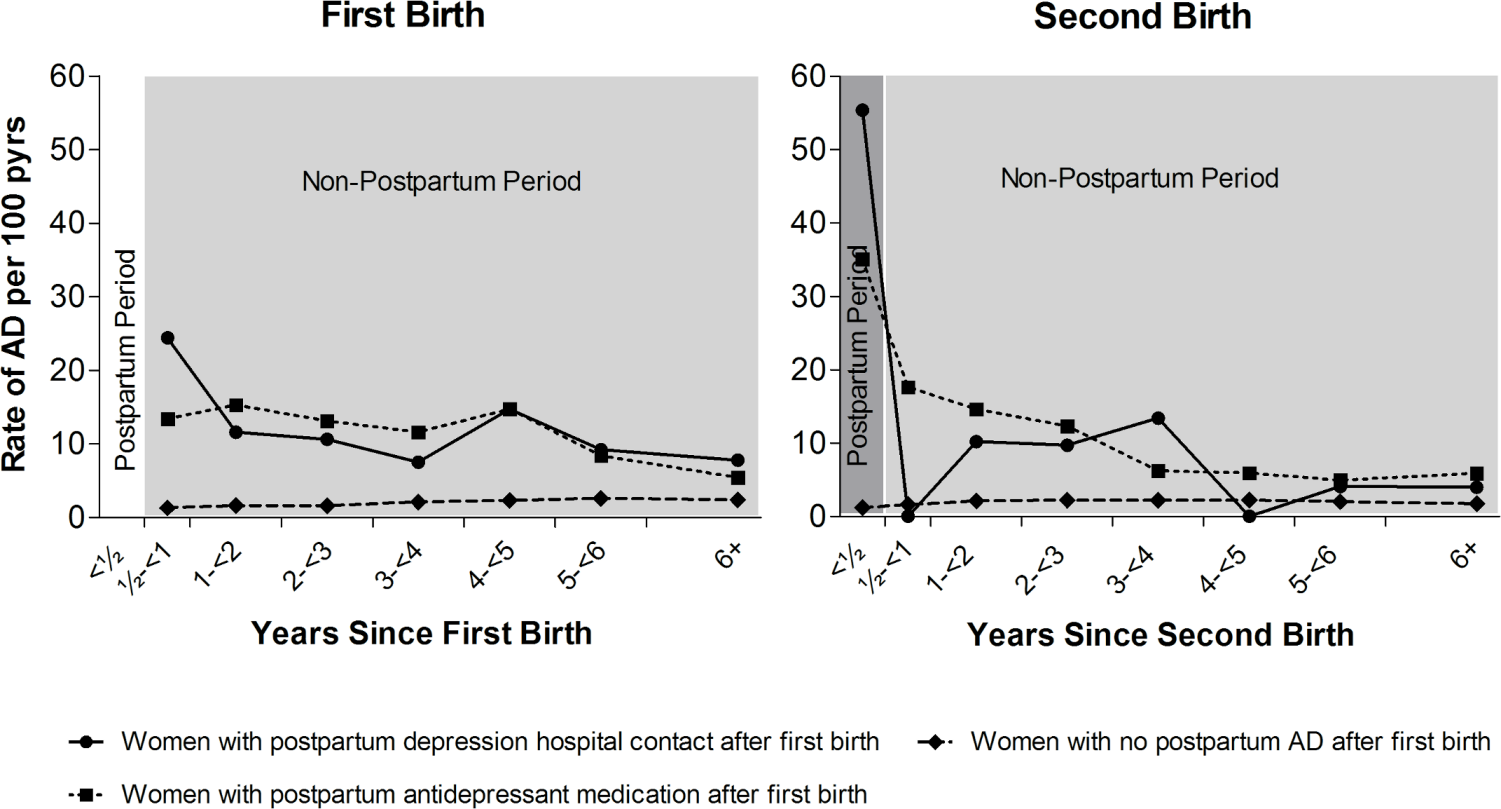
Rates of non-postpartum and postpartum affective disorder (AD), depending on postpartum AD history. Left: First-birth rates of non-postpartum (light grey) AD, depending on postpartum AD history. Right: Second-birth rates of postpartum (dark grey) and non-postpartum (after first 6 months, light grey) AD by number of years since second birth and the women’s history of postpartum AD after first birth. Danish women, 1996–2013, with no psychiatric disorders prior to first birth. pyrs, person-years.

Fig 2 and Table 2 show the rate of non-postpartum AD in women up to 6 years following first birth, depending on postpartum AD history (postpartum antidepressant medication, PPD hospital contact, or no postpartum AD). Table 2 furthermore shows the RR of postpartum AD after second birth and of non-postpartum AD after first and second birth, depending on postpartum AD history.

**Table 2:** Rates and rate ratios (RRs) of non-postpartum and postpartum affective disorder (AD) after first and second birth depending on postpartum AD status after first birth.

Postpartum AD status after first birth	First birth^c^			Second birth^c^					
	Non-postpartum AD			Postpartum AD			Non-postpartum AD			
	*N*	Rate^a^ (95% CI)	RR^b^ (95% CI)	*N*	Rate^a^ (95% CI)	RR^b^ (95% CI)	*N*	Rate^a^ (95% CI)	RR^b^ (95% CI)
Postpartum antidepressant medication	1,820	12.6 (11.6–13.7)	6.2 (5.6–7.9)	621	35.0 (28.7–42.8)	26.9 (21.9–33.2)	491	10.5 (9.0–12.2)	5.0 (4.3–5.8)
PPD hospital contact	382	12.7 (10.7–15.1)	6.6 (5.7–6.7)	122	55.4 (37.7–81.4)	46.4 (31.5–68.4)	86	7.0 (4.7–10.6)	3.5 (2.4–5.3)
No postpartum AD	432,192	1.8 (1.8–1.9)	1 (ref.)	272,452	1.2 (1.1–1.2)	1 (ref.)	262,642	2.0 (2.0–2.0)	1 (ref.)

Danish women, 1996–2013, with no psychiatric disorders prior to first birth.

^a^Per 100 person-years.

^b^Adjusted for year of birth and mother’s age. To evaluate to what degree the rates in women with postpartum antidepressant medication and PPD hospital contact differed, we also calculated the RR between postpartum antidepressant medication (ref.) and PPD hospital contact: After first birth, non-postpartum AD RR = 1.1 (95% CI 0.9–1.3). After second birth, postpartum AD RR = 1.7 (95% CI 1.1–2.7). After second birth, non-postpartum AD RR = 0.7 (95% CI 0.5–1.1).

^c^Total number of person-years (PY): After first birth, non-postpartum period: (1) women with postpartum antidepressant medication, 4,664 PY; (2) women with a PPD hospital contact, 984 PY; (3) women with no postpartum AD, 1,462,799. After second birth, postpartum period: (1) women with postpartum antidepressant medication, 271 PY; (2) women with a PPD hospital contact, 47 PY; (3) women with no postpartum AD, 133,881 PY. After second birth, non-postpartum period: (1) women with postpartum antidepressant medication, 1,624 PY; (2) women with a PPD hospital contact, 328 PY; (3) women with no postpartum AD, 1,383,944 PY.

PPD, postpartum depression.

In total, 434,394 women were eligible for follow-up for non-postpartum AD, of whom 2,202 had had a postpartum AD episode. The rate of a new AD episode did not depend on the type of postpartum AD history, i.e., among women with postpartum antidepressant medication history (*n =* 1,820), the rate of new AD episodes was 12.6 per 100 person-years, and for women with a PPD hospital contact (*n =* 382), the rate was 12.7 per 100 person-years. For women with no postpartum AD history, the corresponding rate was 1.8 per 100 person-years. Adjusted for women’s age and year of birth, and compared to women with no postpartum AD history, women with a postpartum antidepressant medication history and women with a PPD hospital contact had a 6.2 and 6.6 times higher rate of new AD episodes, respectively (see Table 2). The rate of subsequent AD was particularly high in the initial years after the first birth (women with postpartum antidepressant medication, 0.5 to <2 years: 14.6 per 100 person-years; women with PPD hospital contact, 0.5 to <2 years: 16.5 per 100 person-years). The rate decreased with the number of years since the episode (women with postpartum antidepressant medication, 6+ years: 5.4 per 100 person-years; women with PPD hospital contact, 6+ years: 7.8 per 100 person-years), whereas among women with no postpartum AD history, the rate was relatively constant.

Fig 2 and Table 2 also show the rate of postpartum and non-postpartum AD among women giving birth for the second time, dependent on whether the woman was treated for a postpartum AD episode after her first birth (postpartum antidepressant medication, PPD hospital contact, or no postpartum AD). Overall, 273,195 women had a second birth; 743 of these women had had a postpartum episode after their first birth (Table 1)—122 with a PPD hospital contact and 621 with postpartum antidepressant medication. Twenty-one percent of women with a PPD hospital contact after first birth and 15% of women with postpartum antidepressant medication after first birth experienced a recurrent postpartum episode. Out of the 272,452 women with no previous AD, 1,680 (0.6%) women had a first-time episode of postpartum AD. The rate of postpartum AD after the second birth was 1.7 times higher (95% CI 1.1–2.7) among women with a PPD hospital contact after first birth (55.4 per 100 person-years) than among women with postpartum antidepressant medication after first birth (35.0 per 100 person-years). The rate of postpartum AD after second birth for women with no history of postpartum AD was 1.2 per 100 person-years. The rate of AD in the non-postpartum period after the second birth was, except for the period 0.5 to <1 year, similar for all women with postpartum AD after first birth, regardless of treatment regime. After adjusting for year of birth and mother’s age, women with postpartum antidepressant medication after their first birth had a 26.9 times higher rate (95% CI 21.9–33.2) of recurrent postpartum AD after their second birth and a 5.0 times higher rate of AD in general (95% CI 4.3–5.8) in the years following the second birth, compared to women with no postpartum AD history. In comparison, women with a PPD hospital contact after first birth had a 46.4 times higher rate (95% CI 31.5–68.4) of recurrent postpartum AD and a 3.5 times higher rate of AD in general (95% CI 2.4–5.3).

### Supplementary analyses

In additional analyses, we explored how the main results varied by age and year of giving birth (see S2 Text). We found that young (<25 years) primiparous mothers seemed be characterized by a marginally faster treatment period and a higher rate of non-postpartum AD after first birth than primiparous mothers ≥25 years. A relatively smaller proportion of women having a postpartum AD episode in the beginning of the study period (1996–2000) were still in treatment a number of years after the episode compared to women having a postpartum AD episode in the latter part of the study period (2009–2013) (1 year after initiation: 21% versus 32%).

We furthermore tested the sensitivity of the AD definition by varying the length of the postpartum period and the number of prescriptions required to define an AD episode (S2 Text). While altering the above measures obviously changed the estimated incidence of postpartum AD, it did not change the conclusions regarding duration of treatment and recurrence risk. To further examine our outcome definition of AD as a joint measure encompassing both antidepressant medication and hospital contacts, we conducted a number of subanalyses dividing these 2 groups. Overall, dividing the outcome in 3 groups (the main analyses used 2 groups) did not change the results markedly.

We also analyzed the conversion rate of postpartum AD to bipolar AD. With follow-up up to 19 years, we showed that 3.3% of the women with a postpartum AD episode after first birth converted to bipolar AD. Further details on the additional analyses can be found in S2 Text.

The main focus of this study is on women with no prior psychiatric history. However, in order to compare the proportion of women with AD in the postpartum period with existing literature, we also calculated the proportion among “all births,” i.e., the same birth cohort (singleton, first birth 1996–2013) as the main analyses but with no restriction on previous history of mental illness. Among the 920,965 births in the “all births” cohort (i.e., 789,068 births to women with no previous mental illness and 131,897 births to women with previous mental illness), we found 22,251 (2.4%) AD episodes in the postpartum period.

## Discussion

In this nationwide, population-based cohort study, 0.6% of childbirths among women with no prior history of psychiatric disease resulted in a postpartum AD, defined as a prescription fill for antidepressant medication and/or hospital contact for depression during the first 6 months after birth. However, less than 1/3 of the women were still receiving treatment 1 year after treatment initiation. Compared to women with no episode of postpartum AD after their first childbirth, women with postpartum antidepressant medication and PPD hospital contact, respectively, had a 6.2 and 6.6 times increased risk of a non-postpartum AD in the years following first childbirth and a 27 and 46 times higher recurrence rate of postpartum AD following a second birth.

To our knowledge, no other study has specifically addressed the duration of antidepressant use in the postpartum period. We found that a substantial proportion of women filled only 1 prescription for antidepressants, and that less than 28% remained in treatment for 1 year or more. This could reflect that symptoms subsided faster than expected (although a maintenance therapy period of at least 6 months is recommended) [16], or that women stopped treatment due to adverse effects of the medicine or out of concern for the child if they were breastfeeding. If the latter is the case and women dropped out of treatment, we would expect women to relapse and treatment to be restarted for months or years following childbirth. In contrast, the rate of treatment diminishes with time for those who received antidepressant treatment after their first childbirth (Fig 2). Therefore, our finding most likely reflects the transitory nature of PPD. This interpretation is supported by a study that investigated different trajectories of perinatal depressive symptomatology and found 5 different classes, including a “postpartum class,” for which depressive symptomatology resolved after 12–24 months postpartum [13].

Women with a history of PPD have an increased risk of experiencing a recurrence in connection with a subsequent delivery [6,17–19]. The population-based nature of our design allowed us to quantify the recurrence risk as 15% for women with postpartum antidepressant medication after first birth and 21% for women with a PPD hospital contact after first birth, or, in other terms, 27 and 46 times higher, respectively, than for women who did not have a history of postpartum AD after first birth. Thus, women with a PPD hospital contact had an almost twice as high AD recurrence rate compared to women with postpartum antidepressant medication. To the extent that PPD hospital contact is an indicator of a more severe PPD episode compared to medication treatment, the severity of the previous episode seems to significantly influence a women’s risk of a recurrent postpartum AD episode. This finding could perhaps reflect a more proactive treatment strategy among physicians for women with a previous severe episode of PPD (hospital contact), or simply that the more severe the previous PPD episode was, the higher the risk of a recurrent postpartum AD episode—a common predisposing factor underlying the risk of developing PPD in a dose–response relationship.

In the “all births” cohort (not restricting on previous mental history), we found that 2.4% of women had a postpartum AD episode; we considered these likely to have experienced PPD. There is evidence that the majority of PPD episodes are never diagnosed and treated [20,21]. An American study found that only 15% of postpartum women who, according to interview, had experienced a mood disorder during the first year after childbirth had sought help, had been prescribed medications, or had had hospital contact because of their problem [22]. If generally applicable, a postpartum AD risk of 2.4% as measured in our study would correspond to an underlying risk of 16% of PPD, which is consistent with the 10%–15% reported in studies on PPD that have relied primarily on self-reports [1,21,23,24]. A systematic study of 6,790 Danish women who had given birth showed that only 6% had PPD according to the Edinburg Postnatal Depression Scale, so it cannot be ruled out that the prevalence of PPD is lower in Denmark and similar countries with a developed welfare system than in other countries [24,25]. Using the same assumptions for women with no previous mental history, the observed proportion of women receiving postpartum AD treatment of 0.6% corresponds to an underlying risk of 4% of PPD. Episodes captured by treatment status (i.e., medicine use and/or hospital contact) without doubt constitute a group of women likely to be at the more severe end of the PPD spectrum.

Few studies have assessed PPD rate by methods comparable to the present study. Two studies based on the large prospective Danish National Birth Cohort reported a PPD prevalence of 1.6% and 1.8%, respectively, based on prescriptions of antidepressant medication in the first year postpartum [26,27], whereas a Danish population-based cohort study of antidepressant drug use from 12 months prior to childbirth to 12 months postpartum reported a prevalence of 3.2% [28]. However, none of the aforementioned studies were restricted to women free of psychiatric disease prior to enrolment, and this may at least in part, along with the differences in the length of the postpartum period, explain the lower PPD incidence in our study. A population-based study from Finland on hospitalization only for a postpartum period of 6 weeks showed a prevalence of PPD of 0.1% among women with no history of depression [29]. Interestingly, a study from the UK examining the recurrence of PPD showed that for women with a de novo PPD episode, i.e., no previous depressive events, the risk of further episodes of PPD, but not non-postpartum depression, was increased compared to women for whom the PPD episode was a recurrence of depression [14].

Major strengths of this study include its population-based prospective design, its large size, and the utilization of high-quality Danish national registers. Healthcare in Denmark is free of charge, which ensures that all residents, irrespective of economic status, receive appropriate treatment. The information used was mandatorily reported to the national registries and did not rely on self-report. This allowed us to estimate absolute AD rates with little bias. To our knowledge, this is the first population-based cohort study to present risk estimates for PPD in a population initially free of psychiatric problems, and to provide figures for the duration of treatment and rate of recurrence after a second birth.

While some of the strengths of this study are based on the use of the national registers, so are some of the limitations. The use of register data on antidepressant prescriptions to define AD implies that the women in this study do not necessarily fulfill the DSM-IV diagnostic criteria for PPD, as this type of medication is often prescribed for indications other than depression, such as anxiety and obsessive-compulsive disorder, which is why we refer to these women as being treated for an AD. However, results from a subanalysis including only hospital admissions showed that the overall conclusions regarding recurrence risk were not markedly different for this subgroup (see S2 Text). Psychiatric disorders in the postpartum period, especially in the early postpartum period, may be a marker of possible underlying bipolarity [30]. In our cohort of women with no psychiatric episodes prior to postpartum AD and with a 19-year follow-up period, only 3.3% of postpartum AD episodes later converted to bipolar illness (S2 Text).

Different assumptions made in this study are debatable; we have investigated these further in supplementary analyses (S2 Text). The length of the postpartum period is debatable [31]; we chose to employ a 6-month postpartum period [32] in the main analyses, and accompany these with subanalyses using a 3-month and 12-month postpartum period. Further, we also conducted separate analyses for PPD hospital contacts only, use of antidepressant medication only, and women who filled at least 2 prescriptions of antidepressants rather than only 1. The supplementary analyses did not change the overall conclusions regarding the duration of treatment and recurrence risk (S2 Text).

The RRs of recurrence in Table 2 are all adjusted for year of birth and mother’s age, and in Table 1 also for parity. We did not have information on personal traits or sociodemographic variables. Thus, it is possible that some of the RRs for recurrence might reflect personal propensity towards AD.

To conclude, in this study, an antidepressant treatment or depression diagnosis through hospital contact within 6 months after childbirth was observed in 0.6% of childbirths among women with no previous psychiatric history. The estimated recurrence risk of postpartum AD was 15% for women with postpartum antidepressant medication after first birth and 21% for women with a PPD hospital contact after first birth, and the observed risk of treatment for depression remained increased for several years. However, treatment duration for the majority of women in the study was short. These population-based figures provide valuable guidance to physicians treating women with PPD. The study documents the existence of a group of patients who experience elevated rates of subsequent depression and PPD following an initial postpartum AD episode. It underlines the seriousness of single initial episodes and highlights the necessity of both primary and secondary preventive measures, of which several exist [33,34].

## Supporting information

S1 STROBE Checklist (DOC)

S1 Text Description of registries. (DOCX)

S2 Text Supplementary and sensitivity analyses. (DOCX)

## Abbreviations

AD: affective disorder
DNPR: Danish National Prescription Registry
NPR: National Patient Registry
PCRR: Psychiatric Central Research Register
PPD: postpartum depression
RR: rate ratio
