# Supplementary material for: Risk, treatment duration, and recurrence risk of postpartum affective disorder in women with no prior psychiatric history: A population-based cohort study
Source: PLoS Med. 2017 Sep 26;14(9):e1002392. doi: 10.1371/journal.pmed.1002392 (PMC5614423; doi:10.1371/journal.pmed.1002392)
Supplement: S1 Text — (DOCX) [file pmed.1002392.s002.docx]

Since April 1968, the Danish Civil Registration System (CRS) has registered demographic and vital status information on all persons residing in Denmark, using the unique personal identification number (PIN) assigned to each Danish resident [1]. The PIN permits complete follow-up of persons living in Denmark and accurate linkage of individual-level information from Denmark’s national population-based registers.

The Danish Psychiatric Central Research Register (PCRR) holds information on all admissions to psychiatric hospitals in Denmark since 1969 according to the International Classification of Diseases (8th revision [ICD-8] 1977–1993; ICD-10 from 1994). Furthermore, it contains information on psychiatric contacts at outpatient clinics since 1995 [2].

The Danish National Patient Register (NPR) has recorded information on all hospitalizations in Denmark since 1977 and all outpatients and consultant visits since 1995. As the PCRR, it uses ICD-8 codes from 1977–1993 and ICD-10 codes from 1994 [3].

Danish National Prescription Registry (DNPR) was established in 1994 and is considered complete from January 1995. Each record includes the personal identification number, dispensing date, anatomic therapeutic chemical (ATC) code, and number of defined daily doses in the prescription [4].

**References:**

1. Pedersen CB. The Danish Civil Registration System. ScandJPublic Heal. 2011;39(1651–1905):22–5.

2. Mors O, Perto GP, Mortensen PB. The Danish Psychiatric Central Research Register. ScandJPublic Heal. 2011;39(1651–1905):54–7.

3. Lynge E, Sandegaard JL, Rebolj M. The Danish National Patient Register. ScandJPublic Heal. 2011;39(1651–1905):30–3.

4. Kildemoes HW, Sorensen HT, Hallas J. The Danish National Prescription Registry. ScandJPublic Heal. 2011;39(1651–1905):38–41.
