# Supplementary material for: Risk, treatment duration, and recurrence risk of postpartum affective disorder in women with no prior psychiatric history: A population-based cohort study
Source: PLoS Med. 2017 Sep 26;14(9):e1002392. doi: 10.1371/journal.pmed.1002392 (PMC5614423; doi:10.1371/journal.pmed.1002392)
Supplement: S2 Text — (DOCX) [file pmed.1002392.s003.docx]

**Stratification according to mother’s age and year of birth**

In general, young (<25y) primiparous mothers seems be characterized by a marginally faster treatment period than primiparous mothers above 24 years. However, young primiparous mothers with a postpartum affective disorder (AD) episode have a comparatively higher rate of non-postpartum AD after first birth.

A relatively lower proportion of women having a postpartum AD episode in the beginning of the study period (1996-2000) were still in treatment a number of years after the initiation when compared to women having a postpartum AD episode in the latter part of the study period (2009-2014) (1y after: 21% vs 32%). The rate ratios of AD after first and second birth were higher among women giving birth in the beginning of the period compared to later. This is largely due to the low rates of AD among women with no postpartum AD history in the beginning of the study period.

**Postpartum affective disorder (AD) and grace period**

First, we only included women who either were admitted to a hospital or received *two or more* prescriptions of antidepressants, as opposed to one or more in the main analysis. One and four years after the first postpartum AD episode 31.5% and 6.2%, respectively, of primiparous mothers remained in treatment. Primiparous women with a postpartum AD episode had an adjusted 6.5 times higher rate (95% CI=6.2-7.1) of a non-postpartum AD episode in the years after the first birth, and a 36.2 (95% CI=29.8-44.0) times higher rate of a recurrent postpartum AD episode subsequent to a second birth compared to women with no postpartum AD episode after first birth.

In the main analysis, an AD episode was defined as a depressive episode within six months after childbirth. However, in sub-analyses we applied a three months and a twelve months definition instead. With a three months postpartum period, the recurrence rate of postpartum AD after second birth increased markedly for women with a previous episode of postpartum AD (52.8 per 100 person-years compared to 38 per 100 person-years when employing a six months postpartum period). The rest of the results did not change markedly from the main analysis.

From the total number of postpartum AD episodes in first-time mothers, 85% of the cases were identified solely by use of antidepressants, 12% where identified both via prescriptions and hospital contact(s) and 3% were identified solely by one or more hospital contact(s). Consequently, we conducted sub-analyses of the recurrence rates where we split both the exposure (postpartum AD after first birth) and the outcome (postpartum AD after second birth) in three groups; Postpartum antidepressant medication/Postpartum depression hospital contact/No postpartum AD. Overall, dividing the outcome in three groups (the main analyses used two groups) did not change the results markedly. However, splitting the data in this manner gave us very few cases in the only “Postpartum depression hospital contact” groups.

Lastly, we investigated the use of a three months grace period between consecutive prescriptions in order to define a continuous postpartum AD episode in the main analyses. We chose to explore two alternative lengths of grace periods, a null and a six months grace period, respectively. Assuming no grace period (null months) between two consecutive prescriptions did change the results markedly from the main analyses. One and four years after the initiation of treatment 6.6% and 0.6%, respectively, remained in treatment. Primiparous women with a postpartum AD episode had a 20 times higher rate (95% CI=19.2-21.5) of treatment for depression in the years after the first birth, and a 14 times higher rate (95% CI=11.0-18.3) of a recurrent postpartum AD episode subsequent to a second birth than women with no postpartum AT episode at first birth. Using a six months grace period instead, the proportion of primiparous women in treatment by first and fourth year after the initiation of treatment was 32.8% and 8.2%, respectively. For women with a postpartum AD after the first birth the rate of a new non-postpartum episode was 9.3 per 100 person-years, whereas the rate for women with no such history was almost the same as in the main analysis. The recurrence rate of postpartum AD subsequent to a second birth was 28 times higher (95% CI 24.4-34.2) in women with a previous postpartum AT compared to women with no previous postpartum AT.

**Conversion rate to bipolar affective disorder**

To evaluate the risk of a depressive episode later proving to be an underlying bipolar illness we illustrated the probability of converting to bipolar disorder among women with a postpartum AD episode after first birth by Kaplan Meier estimates. With follow-up up to 19 years, we showed that 3.3% of the women with a postpartum AD episode after first birth converted to bipolar affective disorder. According to the timing of the postpartum AD onset (i.e. onset 1-30d post-partum vs. 31-365d post-partum) 2.3% and 3.4% of the cases converted to bipolar affective disorder, respectively. Because of a limited number of cases we were not able to stratify the analysis according to the severity of the episode (i.e. prescriptions only vs. hospital admissions).
